# Effectiveness of the Apparent Diffusion Coefficient for Predicting the Response to Chemoradiation Therapy in Locally Advanced Rectal Cancer: A Systematic Review and Meta-analysis

**DOI:** 10.1097/MD.0000000000000517

**Published:** 2015-02-13

**Authors:** Haiting Xie, Tao Sun, Ming Chen, Hao Wang, Xin Zhou, Yunkai Zhang, Huanhong Zeng, Jilian Wang, Wei Fu

**Affiliations:** From the Department of General Surgery (MX, TS, XZ, YZ, HZ, JW, WF), Peking University Third Hospital; Department of Radiology (MC), Peking University Third Hospital; and Department of Radiation Oncology (HW), Peking University Third Hospital, Beijing, China.

## Abstract

The efficacy of the different apparent diffusion coefficients (ADCs) in predicting different responses to preoperative chemoradiation therapy (CRT) in patients with locally advanced rectal cancer (LARC) is controversial. We did this meta-analysis to evaluate the efficacy of different ADCs predicting different responses to CRT in patients with LARC.

We systematically searched the MEDLINE, Embase, and Cochrane Library databases for articles published from January 1, 1990, to June 3, 2014. Pooled estimates were calculated using a bivariate random-effects model for the ADCs before and after CRT (pre- and post-ADC), as well as the change between the pre- and post-ADC (ΔADC). The values of the 3 ADCs for judging different response endpoints, which were defined according to the tumor grading (TRG) system and downstaging of T (tumor) or N (nodal) stages (TN downstaging), were assessed.

We included 16 studies with a total of 826 patients. The sensitivity, specificity, DOR, and AUC were 75% (95% CI 57%–87%), 70% (95% CI 50%–84%), 6.81 (95% CI 2.46–18.88), and 0.79 (95% CI 0.75–0.82), respectively, for the pre-ADC in predicting a good response; 76% (95% CI 63%–85%), 87% (95% CI 78%–92%), 20.68 (95% CI 11.76–36.39), and 0.89 (95% CI 0.86–0.91), respectively, for the post-ADC; and 78% (95% CI 65%–87%), 77% (95% CI 62%–87%), 11.82 (95% CI 4.65–30.04), and 0.84 (95% CI 0.81–0.87), respectively, for the ΔADC. The post-ADC demonstrated the highest specificity and DOR (*P* < 0.001), although sensitivity did not differ between the 3 types of ADC (*P* = 0.380, 0.192, and 0.214). For predicting a pathological complete response (pCR), the post-ADC had the highest specificity (*P* < 0.001and 0.030) but lowest sensitivity (*P* < 0.001). The ΔADC had the highest DOR; however, this difference was not statistically significant (*P* = 0.146).

The ADC is a reliable and reproducible measure and could serve as a promising noninvasive tool for evaluating the response to CRT in patients with LARC; the post-ADC and ΔADC are particularly promising. The ΔADC had the highest diagnostic performance to predict a pCR compared with the pre-ADC and post-ADC. The value of the ADCs to predict T or N downstaging requires further investigation.

## Abstract 2

Supplemental Digital Content is available in the text

## INTRODUCTION

Preoperative chemoradiation therapy (CRT) is a standard treatment for patients with locally advanced rectal cancer (LARC). However, individuals’ responses to CRT vary widely. Only 50% to 60% of patients are downstaged, with approximately 20% of patients exhibiting a pathological complete response (pCR).^1–3^ Different CRT responses correlate with different long-term outcomes in patients with rectal cancer. Park et al found that the 5-year recurrence-free survival rates were 90.5%, 78.7%, and 58.5% for patients with complete, intermediate, and poor responses, respectively.^2^ Therefore, it is necessary to find a favorable tool for predicting the response to CRT, thus allowing for early surgery in poor responders and a wait-and-see nonoperative approach in complete responders.

Magnetic resonance imaging (MRI) with diffusion-weighted imaging (DWI), a type of functional MRI, is widely used to differentiate between different degrees of response to CRT and has proven to be more valuable than morphological MRI because it can assess the biological characteristics of tissues and quantify the apparent diffusion coefficient (ADC).^4–6^ However, the diagnostic performances of ADCs before and after CRT (pre- and post-ADC), as well as the change between pre- and post-ADC (ΔADC), differed in previous studies.^7^ In particular, the pre-ADC varied with some studies having demonstrated that the pre-ADC measurement is lower in good responders,^8–12^ but other studies identifying no difference in the pre-ADC measurements between good and poor responders.^13–17^ The difference in ΔADC was smaller and most studies reported that the absolute and relative ΔADC values were increased in good responders; however, no significant difference between good and poor responders was still noted in a few studies.^13,16^ Additionally, there is no consistency regarding the endpoint of pathologic response after CRT when assessing the predictive value of the 3 ADCs. Different endpoints, such as the tumor grading (TRG) system and downstaging of T (tumor) or N (nodal) stages (TN downstaging), have been used in different studies, making the outcomes more controversial.^8^

A previous meta-analysis summarized the value of DWI in predicting CRT responses for LARC patients via subgroup analyses.^18^ However, the authors did not evaluate the diagnostic performances of pre-ADC, post-ADC, and ΔADC. Additionally, the values of ADCs in determining different endpoints of response were not highlighted. Furthermore, many studies with different conclusions have been published; thus, it is imperative to perform another meta-analysis. According to our knowledge and experience, we hypothesized that ΔADC may have the highest value in different endpoints, while pre-ADC was on the contrary. In order to confirm it, we made this meta-analysis to assess the value of 3 ADCs in patients demonstrating a good response to CRT, and also to assess the value of these ADCs in judging different response endpoints.

## METHODS

The reporting of the present review adhered to the preferred reporting of items for systematic reviews and meta-analyzed (PRISMA) statement.^19^

### Criteria for Study Eligibility

Studies were included if they fulfilled the following criteria: MRI with DWI was used to predict the CRT response in LARC patients; the histopathological therapeutic response was used as the reference standard; original articles (if data were used in more than 1 article, only the newest paper was included); original primary data were available to extract or reconstruct 2 × 2 contingency tables. If this information was lacking, the authors were contacted with requests for the information. Studies with any of the following features were not eligible for inclusion: non-English articles; animal experiments; and reports available only as abstracts, reviews, lectures, letters to the editor, and articles published in books.

### Literature Search and Data Extraction

A literature search was performed for relevant publications published between January 1, 1990, and June 3, 2014, using MEDLINE, Embase, and Cochrane Central. Keywords, such as rectal cancer, preoperative chemoradiation therapy, response, and magnetic resonance imaging were used (Appendix A, http://links.lww.com/MD/A201). We also conducted searches in Google Scholar (the first 100 results only), the WHO Web site, and IndMED and African Index Medicus databases. Reference lists of the retrieved articles were manually searched to identify relevant studies. Two veteran reviewers (X.Z. and Y.Z.) independently read all the titles and abstracts of all studies, using predefined criteria. The full text was read if the titles and abstracts did not provide enough information. Studies were excluded based on the above criteria.

### Study Population

The study population was as follows: patients with biopsy-proven rectal cancer, MRI with DWI was performed, tumor staging is T3–4NxM0 by imaging, treated with a long course chemoradiation therapy prior to surgical resection, and men and women, with no restriction on age or country.

### Definition of Endpoint

Different endpoints of pathologic response after CRT were assessed in the studies. Most studies used the TRG system as the endpoint, but some studies used TN downstaging. In the studies using the TRG system, some authors defined patients with a pCR (TRG1 in Mandard classification^20^ and TRG4 in Dworak classification^21^) as good responders, but some authors also demonstrated that patients with TRG2 (Mandard classification) or TRG2–3 (Dworak classification) were also good responders. For consistency, we defined patients with TN downstaging, TRG1–2 (Mandard classification), or TRG2–4 (Dworak classification) as good responders and other patients as poor responders. Finally, in this meta-analysis, 5 endpoints were used to assess the predictive value of 3 ADCs: good response, pCR, T downstage, N downstage, and TRG 1–2 (Mandard classification, TRG 2–4 in Dworak classification).

### Assessment of Methodological Quality

The quality of the included studies was assessed using the Quality Assessment of Diagnostic Accuracy Studies-2 (QUADAS-2) as recommended by the Cochrane Collaboration.^22^ For the purpose of this meta-analysis, we added 1 signaling question in the domain of Patients Selection and 2 signaling questions in the domain of Index Text. The description of each item was listed in Table 1. Two reviewers (Xin Zhou, MD, and Yunkai Zhang, MD) evaluated each study independently. Disagreements were resolved by discussion with a third reviewer (Wei Fu, MD) who was blinded to the assessments of the other 2 reviewers.

**TABLE 1:**
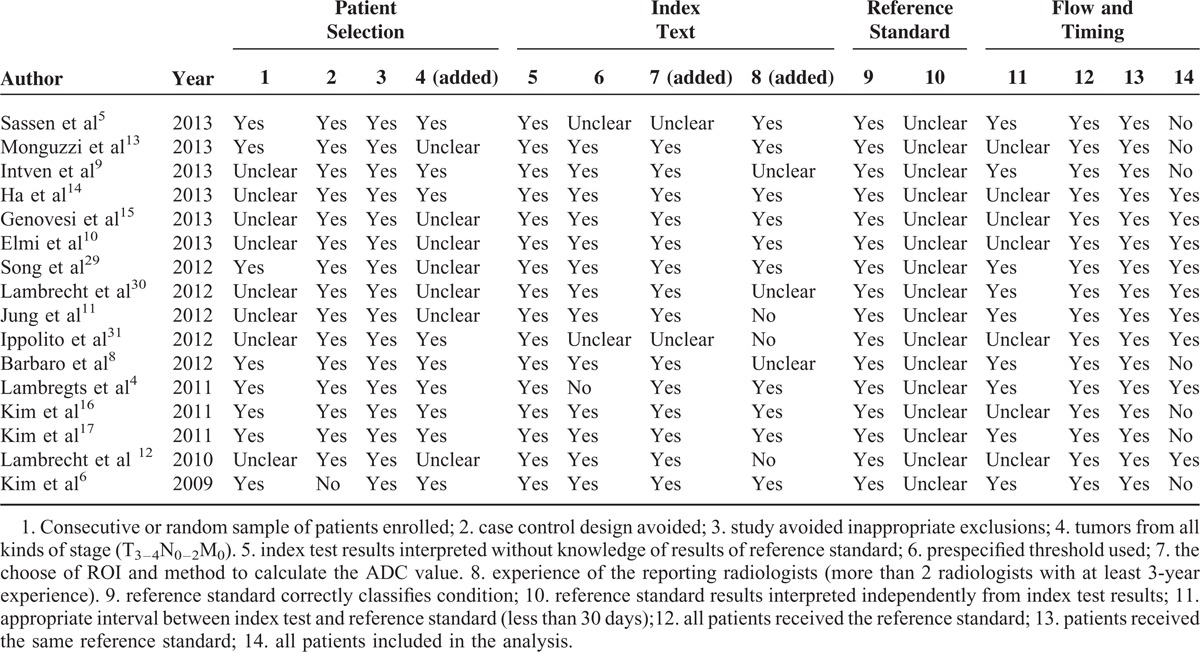
The Quality Assessment of Diagnostic Accuracy Studies-2 Tool for Quality Assessment of the Included Studies

### Statistical Analysis

In this meta-analysis, a bivariate random-effects model was used to generate the summary estimates of the sensitivity, specificity, and diagnostic odds ratio (DOR)^23,24^ of the 3 ADC values (pre-ADC, post-ADC, and ΔADC) in judging the 5 different response endpoints mentioned above (if the subgroup analysis included 4 or fewer studies, the DerSimonian–Laird model was used). Outcomes were illustrated in the form of forest plots and tables. Additionally, we generated a hierarchical summary receiver operating characteristic curve (HSROC) that plotted the summary estimates of the sensitivity and specificity with 95% confidence intervals (CI)^25,26^ and area under the curves (AUC) was calculated. The pooled sensitivity, specificity, DOR were compared using a t-test was performed, and the *P* value was calculated. However, AUCs could not be compared using a statistical test because they were calculated using HSROCs.

Subgroup analyses according to the different race of the patients were performed (Caucasians vs Asians). We also performed a leave-one-out sensitivity analysis by omitting individual studies one at a time from the meta-analysis. After excluding 1 study, if the pooled outcomes were not within the 95% CI of the original pooled outcomes, the study was considered to be influential. Kappa values (0–0.2 poor, 0.21–0.4 fair, 0.41–0.6 moderate, 0.61–0.8 good, and 0.81–1.0 excellent agreement) were calculated to evaluate interobserver variability when assessing the quality of the included studies. The inconsistency index (*I*^2^) test was used to estimate the heterogeneity between each study.^27^ The publication bias was assessed by producing a Deeks funnel plot and asymmetry test, and publication bias was considered to be present if there was a nonzero slope coefficient (*P* < 0.05).^28^

We used Stata SE version 12 and Review Manager version 5.2 for all statistical analyses. All tests were 2 sided, and *P* < 0.05 was considered statistically significant. The summary estimates of the sensitivity, specificity, and DOR were produced with 95% CIs. Ethical approval was not required, as all analyses were based on previously published studies.

## RESULTS

**FIGURE 1:**
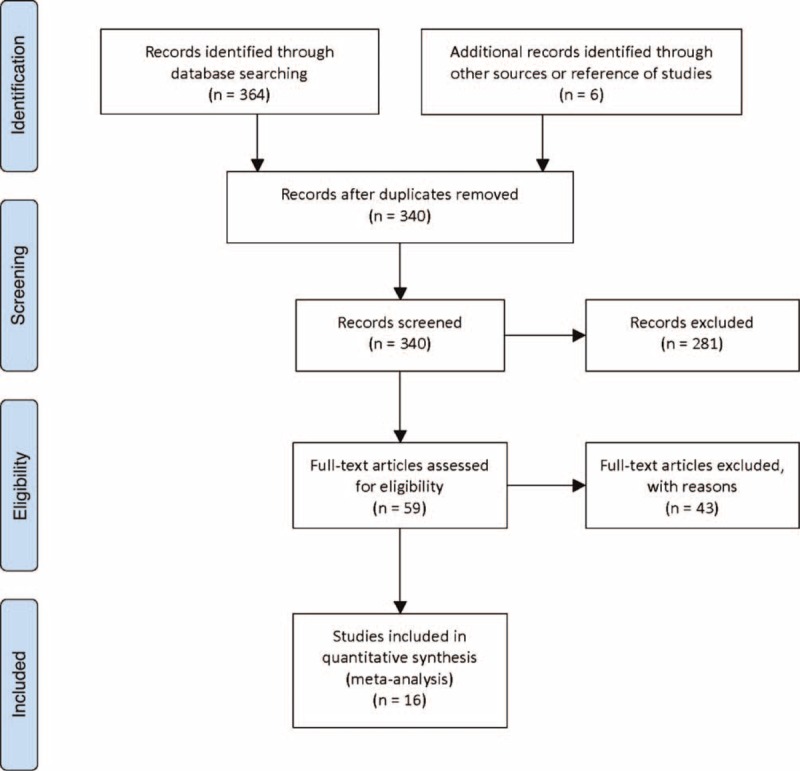
Flow chart of the search results.

The literature search identified 370 references, including 141 studies from MEDLINE, 216 from Embase, 7 from Cochrane Central, and 6 from reference lists. Thirty studies were duplicated, and 281 were excluded on the basis of their titles and abstracts. Then, 59 full-text articles were reviewed to gather more information. After assessing the 59 full-text articles, 6 studies were excluded due to lack of 2 × 2 contingency tables, 33 were excluded because the MRI scan lacked DWI, 3 were excluded because ADC values were not used as index text, and 1 was excluded because the number of patients was less than 10. Finally, 16 studies involving 826 patients were considered relevant for this meta-analysis (Figure 1).^4–6,8–17,29,30^

Table 2 presents the main characteristics of the 16 included studies. There were 9 prospective and 7 retrospective studies. Eight of 16 studies drew regions of interest (ROIs) on the entire tumor volume, 6 drew ROI on a single or 3 sections of the ADC map. Patients in 6 studies were Asians, and the remainder of the patients were Caucasians.

**TABLE 2:**
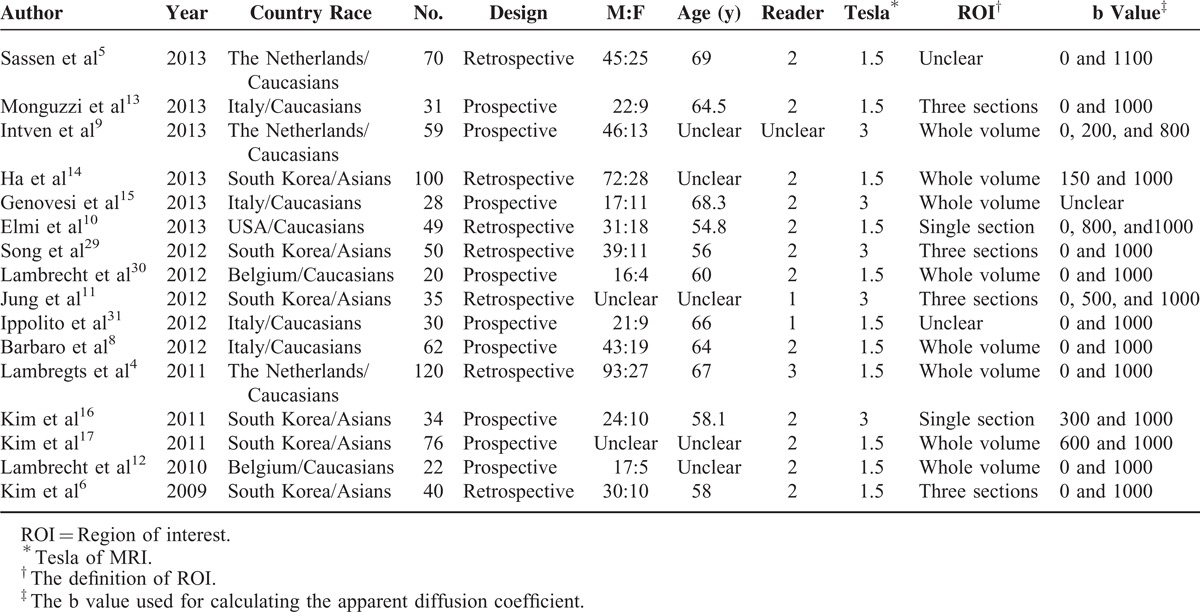
Characteristics of the Included Studies

**FIGURE 2:**
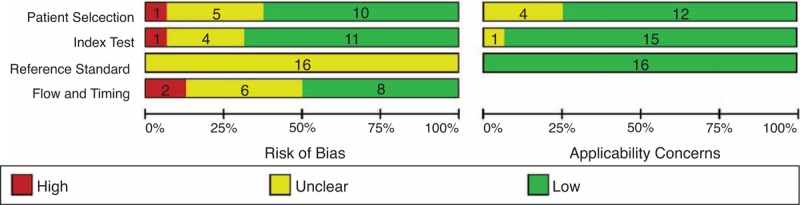
Risk of bias and applicability concerns graph: The reviewers’ judgments about each domain are presented as percentages across the included studies. This graph shows that the quality of the included studies is moderate. Although a few of studies had high risk in each Domain, the studies with unclear risk in each Domain were numbers.

The quality of the included studies is moderate. Although a few of the studies had a high risk in each domain, the studies with unclear risk in each domain were numerous (Figure 2 and Table 1). In the domain of Patient Selection, the risk of bias was high or unclear in 6 studies because it was unclear whether these were consecutive studies or if they included tumors from all stages (T_3–4_N_0–2_M_0_). In the domain of Index Text, the risk of bias was unclear in all of the included studies because none of the studies mentioned whether pathologists were blinded to the information obtained by the radiological analyses. In the domain of Flow and Timing, only 8 studies exhibited a low risk of bias. The main reason for this risk of bias was an unclear time interval between the MRI scan and surgery; in addition, some studies did not include all patients in the final analysis. The kappa value for the interobserver agreement between the 2 veteran reviewers (Xin Zhou, MD, and Yunkai Zhang, MD) was good (κ = 0.656) when assessing the quality of the included studies, but there were still disagreements in 33 judgments (224 judgments total, 14 in each included study). The disagreements were mainly in the domain of Patient Selection, and secondary in the domain of Flow and Timing.

Five endpoints were used to assess the predictive value of 3 ADCs: good response, pCR, T downstaging, N downstaging, and TRG 1–2 (Mandard classification, TRG 2–4 in Dworak classification). The numbers of studies that used the above 5 endpoints were 16, 11, 6, 1, and 6, respectively. Six studies used more than 1 endpoint, and 4 studies had 2 or more reviewers. Not all of the studies provided the 2 × 2 contingency tables for each endpoint. Therefore, the final numbers of studies used to judge these endpoints were 16, 11, 5, 1, and 3, respectively (Appendix B, http://links.lww.com/MD/A201). Because only 1 study^20^ used N downstaging as the endpoint, this endpoint was not analyzed in the meta-analysis. The numbers of studies used to judge the remaining 4 endpoints in different ADC types (pre-ADC, post-ADC, and ΔADC) are listed in Table 3.

**TABLE 3:**
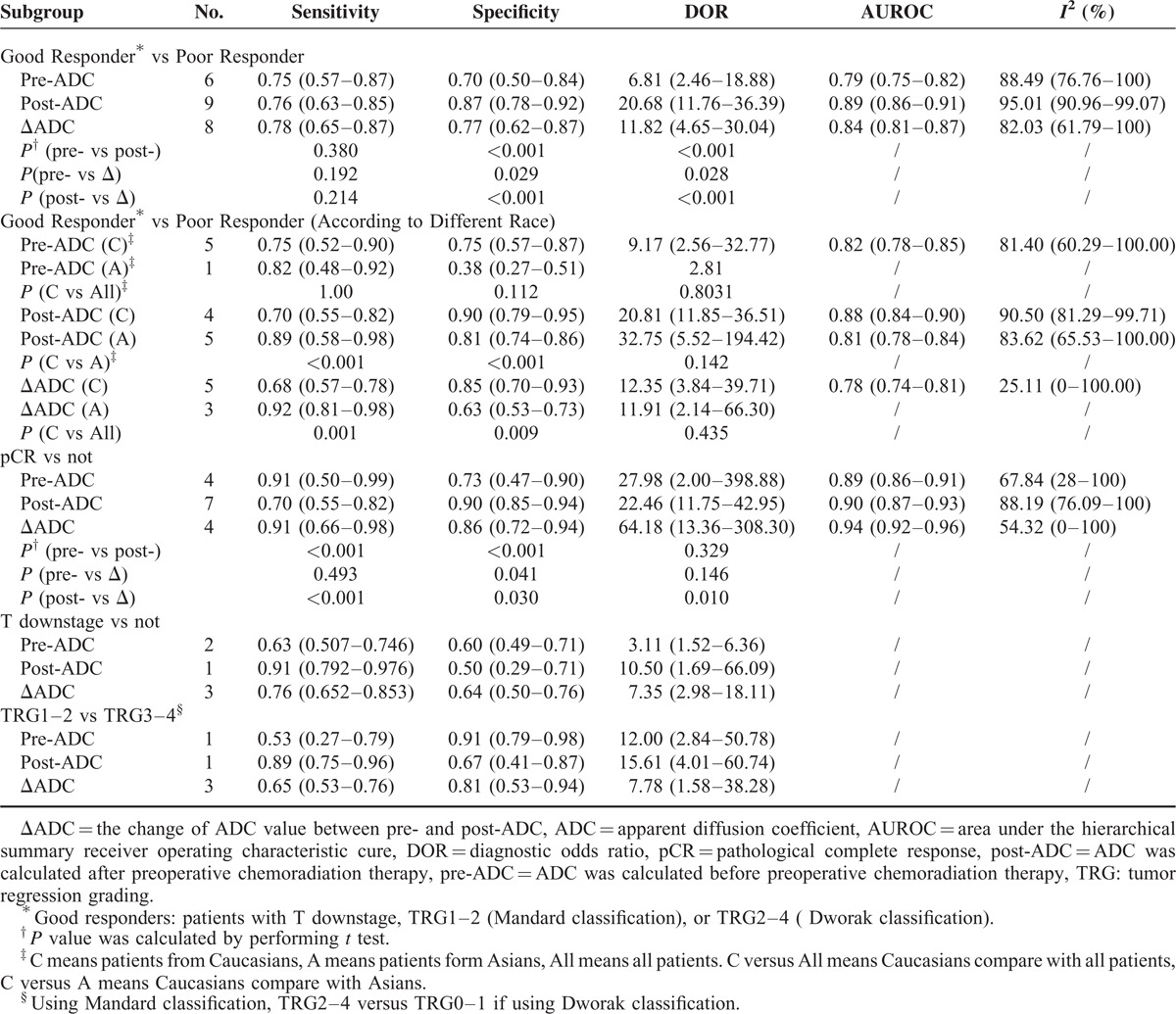
Pooled Estimates of Diagnostic Accuracy of ADC in Different Subgroups

**FIGURE 3:**
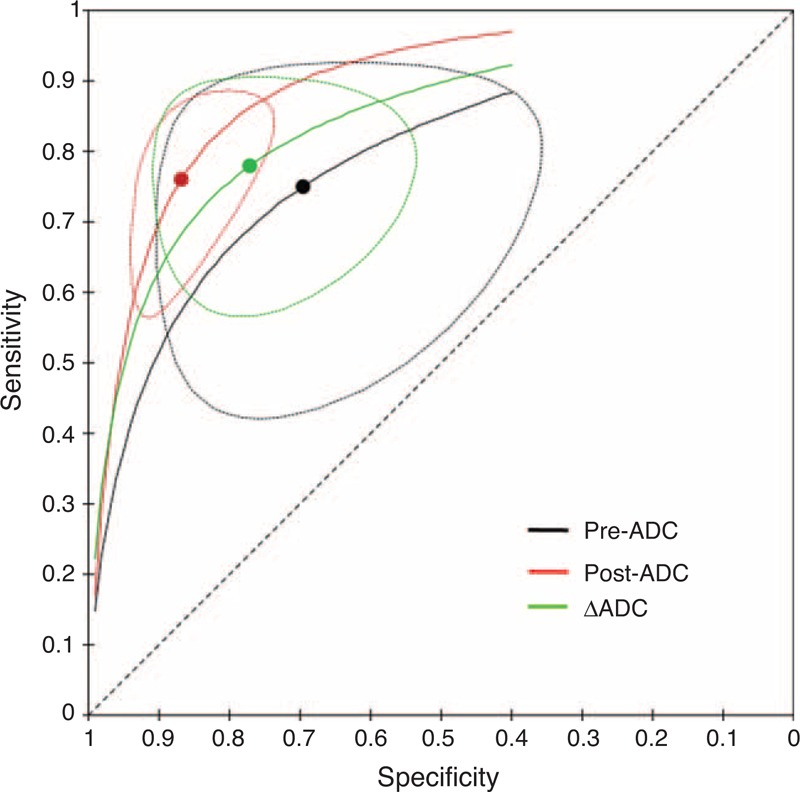
Hierarchical summary receiver operating characteristic curve of the 3 apparent diffusion coefficients (ADCs) for predicting a good response to chemoradiation therapy in patients with locally advanced rectal cancer: 3 cures with 95% confidence intervals (dashed line) were provided for pre-ADC, post-ADC, and ΔADC. Each confidence interval has a summary point (thick points), which represents the most likely values of the true summary sensitivity and specificity.

Table 3 indicates that different ADC types exhibited different pooled estimates for predicting a good response. The sensitivity, specificity, DOR, and AUC were 75% (95% CI 57%–87%), 70% (95% CI 50%–84%), 6.81 (95% CI 2.46–18.88), and 0.79 (95% CI 0.75–0.82), respectively, for the pre-ADC; 76% (95% CI 63%–85%), 87% (95% CI 78%–92%), 20.68 (95% CI 11.76–36.39), and 0.89 (95% CI 0.86–0.91), respectively, for the post-ADC; and 78% (95% CI 65%–87%), 77% (95% CI: 62%–87%), 11.82 (95% CI 4.65–30.04), and 0.84 (95% CI 0.81–0.87), respectively, for the ΔADC. The post-ADC demonstrated the highest specificity (*P* < 0.001) and DOR (*P* < 0.001). Though we did not compare AUCs using a statistical text, from results we could found post-ADC had the highest AUC. The sensitivity did not differ for the 3 ADC types (*P* = 0.380, 0.192, and 0.214). Figure 3 presents the HSROC of the 3 ADC types for predicting a good CRT response in LARC patients.

We also performed a subgroup analysis according to the race (Caucasians vs Asians) when evaluate the 3 ADCs in predicting good response. As shown in Table 3, we found that post-ADC has a higher specificity and lower sensitivity (*P* < 0.001) of predicting Caucasian than Asian, but the DOR did not differ between races (*P* = 0.142). The number of studies with Asian patients was only 1 and 3 when assessing the predictive values of pre-ADC and ΔADC, respectively; therefore, we compared Caucasians with all patients. The sensitivity, specificity, and DOR were not different if we excluded Asians (*P* = 1.00, 0.112, and 0.803) for pre-ADC, but the sensitivity was lower and the specificity was higher if we excluded Asians for ΔADC (*P* = 0.001 and 0.009) (Table 3).

The outcomes of the other 3 endpoints were also listed in Table 3. The sensitivity, specificity, DOR, and AUC to predict a pCR were 91% (95% CI 50%–99%), 73% (95% CI 47%–90%), 27.98 (95% CI 2.00–398.88), and 0.89 (95% CI 0.86–0.91) for the pre-ADC; 70% (95% CI 55%–82%), 90% (95% CI 85%–94%), 22.46 (95% CI 11.75–42.95), and 0.90 (95% CI 0.87–0.93) for the post-ADC; and 91% (95% CI 66%–98%), 86% (95% CI 72%–94%), 64.18 (95% CI 13.36–308.30), and 0.94 (95%CI 0.92–0.96) for the ΔADC. The pre-ADC and ΔADC had higher sensitivities compared with the post-ADC (*P* < 0.001); however, the sensitivity did not differ between the pre-ADC and ΔADC (*P* = 0.493). The post-ADC had the highest specificity among the 3 ADCs (*P* < 0.001, 0.041 and 0.030). The DOR was higher in the pre-ADC than in the post-ADC (*P* = 0.010); however, no clear difference was noted between the other 2 groups (*P* = 0.329 and 0.146). The forest plots of 3 ADCs in judging the 2 response endpoints (good responds and pCR) were shown in Appendices C and D, http://links.lww.com/MD/A202. A small numbers of studies used T downstaging, TRG1–2 (Mandard classification), or TRG2–4 (Dworak classification) as the endpoint. The outcomes of the 2 subgroups are listed in Table 3.

**FIGURE 4:**
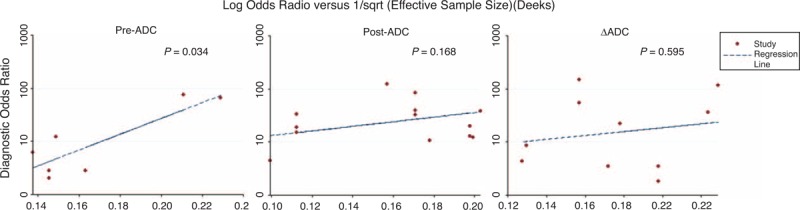
Deeks funnel plot asymmetry test of the apparent diffusion coefficients for predicting a good response to chemoradiation therapy in patients with locally advanced rectal cancer: The plots were determined by linear regression of the inverse root of effective sample sizes on the log diagnostic odds radio. The result was suggestive of publication bias when assessing the value of pre-ADC for predicting a good response (*P* = 0.034), but no strong evidence was produced when assessing the values for post-ADC and ΔADC (*P* = 0.168 and 0.595). ADC = apparent diffusion coefficient, ESS = effective sample size.

We performed a leave-one-study-out sensitivity analysis and Deeks funnel plot asymmetry tests for 1 endpoint (good response), as the numbers of included studies were not large enough when judging the other 3 endpoints. The reliability of the meta-analysis was good, and all the pooled sensitivity and specificity values were within the 95% CI of the original pooled values after excluding 1 study at a time (Appendices E, F, and G, http://links.lww.com/MD/A203). Figure 4 showed the publication bias of the 3 ADCs. There was publication bias when assessing the value of pre-ADC for predicting a good response (*P* = 0.034), but no strong evidence was produced when assessing the predictive values of post-ADC and ΔADC (*P* = 0.168 and 0.595).

## DISCUSSION

In this meta-analysis, we determined that the pre-ADC, post-ADC, and ΔADC were valuable in predicting the response to CRT in LARC.

The post-ADC has been most frequently evaluated as a predictor of response, and nearly all of the studies demonstrated that the post-ADC performs well for selecting good responders and guiding individualized treatment. In our meta-analysis, the post-ADC had the highest DOR and specificity among the 3 ADCs. In good responders, tumors always consist of more necrotic tissue and cells with a variable degree of edema, fibrosis, and inflammation, which are all caused by CRT. These changes are characterized by an increase in the interstitial water content where fewer barriers to diffusion exist, which leads to higher post-ADC.^14,32–33^

Unlike the post-ADC, it was unclear whether the pre-ADC could be a good predictor of response. In our meta-analysis, as a predictor of response, the pre-ADC had less value as a predictor of response than the other 2 ADCs, but was still acceptable. The reason was that the pre-ADC measurements in most poor responders was higher, because high pre-ADC are likely to have tumors with more necrotic tissue and poor cell membrane integrity, the necrotic region is typically poorly perfused, thereby resulting in hypoxic and acidic environments in these areas and leading to higher resistance to CRT^8–12^. However, the measurement may also be high in some good responders,^13–17^ which makes the pre-ADC not valuable enough for predicting CRT responses.

In this meta-analysis, we found that the effectiveness of the ΔADC for predicting the response to CRT was moderate among the 3 ADCs. Because most of studies, but not all, found the absolute and relative ΔADC values were increased in good responders. One reasonable explanation is that more cells in good responders lose their normal structure as a result of interactions with ionizing radiation, resulting in an increase in water diffusion and a greater increase in the ADC value after CRT.^31^ Similar to the increase in the ADC after CRT, an early increase in the ADC during CRT could be a suitable predictor, although the accurate time point for this remains controversial. Cai et al calculated the change in the ADC from 1 to 5 weeks after the beginning of CRT and found a significant increase in the ADC at the end of week 2.^34^ They noted that the significant increase at the second week of treatment correlated with tumor necrosis and apoptosis. Similar results were observed in other studies.^8,35^ However, some studies demonstrated a significant increase in the ADC at the end of the first week.^36,37^ Sun et al demonstrated that vascular endothelial growth factor, which could lead to increased vascular permeability and increased interstitial volume, had a massive release within 1 week after beginning CRT, thereby causing tumor edema and increasing the ADC.^36^

We performed a subgroup analysis according to the race and found that Caucasians have a higher specificity and lower sensitivity (*P* < 0.001) than Asians when assessing the values of post-ADC and ΔADC. This finding was interesting and has not been mentioned in any previous study. The reason is unclear and required more evidence.

The TRG system is widely used in grading the tumor response after CRT because it predicts disease-free survival, metastasis-free survival, and overall survival.^38^ The pCR is a special grade in the TRG system and is defined as the absence of any residual tumor cells in surgical specimens (ypT_0_N_0_). Patients with a pCR after CRT always have a better prognosis than those with other TRG grades. Some researchers have indicated that a wait-and-see nonoperative approach might be safe in patients with a pCR, but longer follow-up intervals, larger samples, and additional careful observational studies are needed.^39^ In this meta-analysis, we determined that the 3 ADCs, especially ΔADC, are all good predictors of a pCR, but some misjudgments remain. The main reason for the misjudgment is that DWI cannot reliably microscopically discriminate residual viable tumor cells from fibrosis, which causes considerable overlap of the ADC values between a pCR and near-pCR.^6,15^ Therefore, although the ADC is a potential quantitative predictor of response, it requires assistance from other tools for the prediction of a pCR, especially for deciding whether patients are eligible for nonoperative management. Some studies used TRG1–2 (Mandard classification) or TRG2–4 (Dworak classification) as an endpoint to assess the value of the ADC in predicting the response to CRT. However, the number of studies is limited, and the predictive values of the 3 ADCs require further investigation.

TN downstaging is not completely concordant with the TRG system and can also predict the prognosis of patients receiving CRT^2^; therefore, this measure was used as an endpoint in a few studies. However, all 3 ADCs have an unfavorable value in predicting TN downstaging. This finding might result from the fact that the initial and postoperative pathological T and N staging remain challenging on MRI, making TN downstaging a less objective definition of a response than pCR.^40–42^

There were several limitations to our study. First and most importantly, substantial heterogeneity was noted. Although we performed subgroup analyses, the heterogeneity remained large. The most important factors causing heterogeneity are the selection of the ROI and b values for calculating the ADC. Of the 16 studies included, 8 used the whole tumor volume as the ROI, whereas 6 studies used only 1 or 3 sections of the tumor and remaining 2 studies were unclear (Table 1). Barbaro et al suggested that the use of the whole tumor is easier and more reproducible. However, the whole tumor always includes areas of necrosis and mucin pools, which could cause an increase in the ADC value and lead to an overestimation of the therapeutic effects.^8^ Other factors, such as variations in the study design, patient characteristics, different parameters of MRI, and the individual differences of radiologists, surgeons, and pathologists, also contributed to the heterogeneity. Second, we restricted our search to studies published in English, which potentially led to language bias. Third, publication bias is also a potential limitation. From the results of Deeks funnel plot asymmetry test we can see that there is publication bias when assessing the value of pre-ADC for predicting a good response (*P* = 0.034) because we cannot totally exclude the possibility that some studies with poor diagnostic performance may have remained unpublished. However, these studies would have to be large to change the results. Fourth, studies that could not be used to extract or reconstruct 2 × 2 contingency tables were excluded. In some of the included studies, the pre-ADC, post-ADC, and ΔADC values were calculated, although the cutoffs of all 3 were not always extracted. These missing data potentially influenced our results.

In conclusion, the ADC is a reliable and reproducible measure and could become a promising noninvasive tool for evaluating the response to CRT in patients with LARC; the post-ADC and ΔADC are particularly promising. Caucasians may have a higher specificity and lower sensitivity than Asians in assessing the predictive values of post-ADC and ΔADC, but requires more evidence. The ΔADC has the best diagnostic performance to predict a pCR compared with the pre-ADC and post-ADC. The value of the ADC to predict TN downstaging, TRG1–2 (Mandard classification), or TRG2–4 (Dworak classification) requires further investigation.
